# Bis{*N*-[bis­(pyrrolidin-1-yl)phosphor­yl]-2,2,2-trichloro­acetamide}di­nitrato­dioxidouranium(VI)

**DOI:** 10.1107/S1600536810006422

**Published:** 2010-02-24

**Authors:** Kateryna O. Znovjyak, Vladimir A. Ovchynnikov, Olesia V. Moroz, Svitlana V. Shishkina, Vladimir M. Amirkhanov

**Affiliations:** a Kyiv National Taras Shevchenko University, Department of Chemistry, Volodymyrska str. 64, 01601 Kyiv, Ukraine; b STC "Institute for Single Crystals", National Academy of Science of Ukraine, Lenina ave. 60, 61001, Khar’kov, Ukraine

## Abstract

The crystal structure of the title compound, [U(NO_3_)_2_O_2_(C_10_H_17_Cl_3_N_3_O_2_P)_2_], is composed of centrosymmetric [UO_2_(*L*)_2_(NO_3_)_2_] mol­ecules {*L* is *N*-[bis­(pyrrolidin-1-yl)phosphor­yl]-2,2,2-trichloro­acetamide, C_10_H_17_Cl_3_N_3_O_2_P}. The U^VI^ ion, located on an inversion center, is eight-coordinated with axial oxido ligands and six equatorial oxygen atoms of the phosphoryl and nitrate groups in a slightly distorted hexa­gonal-bipyramidal geometry. One of the pyrrolidine fragments in the ligand is disordered over two conformation (occupancy ratio 0.58:0.42). Intra­molecular N—H⋯O hydrogen bonds between the amine and nitrate groups are found.

## Related literature

For the synthesis and coordination properties of the ligand *L*, see: Znovjyak *et al.* (2009 ▶). For a structural investigation of *L*, see: Gholivand *et al.* (2006 ▶). For the synthesis and structural investigation of a uranium(IV)-containing complex with a similar ligand, see: Amirkhanov *et al.* (1997 ▶).
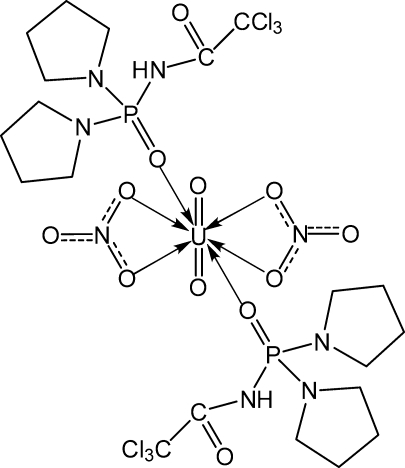


## Experimental

### 

#### Crystal data

[U(NO_3_)_2_O_2_(C_10_H_17_Cl_3_N_3_O_2_P)_2_]
*M* *_r_* = 1091.22
Triclinic, 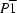

*a* = 9.8292 (7) Å
*b* = 10.3436 (8) Å
*c* = 10.4475 (6) Å
α = 71.905 (6)°
β = 84.391 (5)°
γ = 71.475 (6)°
*V* = 957.34 (11) Å^3^
*Z* = 1
Mo *K*α radiation
μ = 4.80 mm^−1^
*T* = 293 K
0.40 × 0.30 × 0.20 mm

#### Data collection

Oxford Diffraction Xcalibur3 diffractometer
Absorption correction: multi-scan (*CrysAlis RED*; Oxford Diffraction, 2006 ▶) *T*_min_ = 0.250, *T*_max_ = 0.447
22849 measured reflections
5523 independent reflections
5486 reflections with *I* > 2σ(*I*)
*R*_int_ = 0.090

#### Refinement

*R*[*F* ^2^ > 2σ(*F* ^2^)] = 0.034
*wR*(*F* ^2^) = 0.085
*S* = 0.98
5523 reflections
260 parameters
56 restraints
H-atom parameters constrained
Δρ_max_ = 1.93 e Å^−3^
Δρ_min_ = −1.68 e Å^−3^

### 

Data collection: *CrysAlis CCD* (Oxford Diffraction, 2006 ▶); cell refinement: *CrysAlis RED* (Oxford Diffraction, 2006 ▶); data reduction: *CrysAlis RED*; program(s) used to solve structure: *SHELXS97* (Sheldrick, 2008 ▶); program(s) used to refine structure: *SHELXL97* (Sheldrick, 2008 ▶); molecular graphics: *ORTEP-3 for Windows* (Burnett & Johnson, 1996 ▶; Farrugia, 1997 ▶); software used to prepare material for publication: *WinGX* (Farrugia, 1999 ▶).

## Supplementary Material

Crystal structure: contains datablocks I, global. DOI: 10.1107/S1600536810006422/dn2539sup1.cif

Structure factors: contains datablocks I. DOI: 10.1107/S1600536810006422/dn2539Isup2.hkl

Additional supplementary materials:  crystallographic information; 3D view; checkCIF report

## Figures and Tables

**Table 1:** Hydrogen-bond geometry (Å, °)

*D*—H⋯*A*	*D*—H	H⋯*A*	*D*⋯*A*	*D*—H⋯*A*
N1—H1⋯O5	0.86	2.13	2.877 (4)	145

**Fig. 1.**
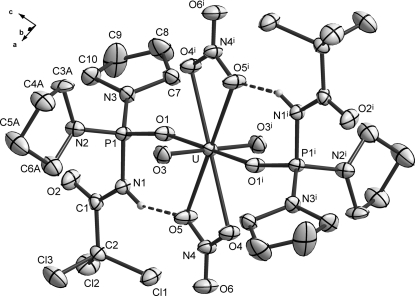
View of the centrosymmetric molecule of 1 with atom numbering scheme. Displacement ellipsoids are drawn at 30% probability level. Hydrogen atoms not involved in hydrogen bonding omitted for clarity. [Symmetry code: (i) -x+1, -y+1, -z+1]

## supplementary crystallographic information

### Comment

As a part of our study of coordination compounds based on carbacylamidophosphates with C(O)NHP(O) structural fragment we obtained the title compound UO_2_(*L*)_2_(NO_3_)_2_ (*L* = 2,2,2-trichloro- *N*-[di(1-pyrrolidinyl)phosphoryl]acetamide) (1), and solved its crystal structure. It was shown previously that *L* is able to form complexes with lanthanides (Znovjyak *et al.*, 2009).

The crystal structure of 1 is made of centrosymmetric molecules UO_2_(*L*)_2_(NO_3_)_2 _and uranium is located on an inversion center. The analysis of the bond lengths and angles of 1 indicates that the coordination polyhedra of the uranium ions are slightly distorted hexagonal bipyramids. The axial vertices are occupied by two oxido ligands while six oxygen atoms of monodentate coordinated *L* ligands and bidentate coordinated nitrate groups lie in the equatorial plane. The nitrate groups additionally form intramolecular hydrogen bonding with the hydrogen atoms of the N—H groups of the *L* ligands (Table 1). In the crystal structure of the complex, the phosphoryl and carbonyl groups are in the *trans* position to each other which was early observed in the structure of the free *L* (Gholivand *et al.*, 2006) and similar complex with uranium ion (Amirkhanov *et al.*, 1997). It was shown that 2,2,2-trichloro-*N*-[di(1-pyrrolidinyl)phosphoryl]acetamide aggregates into non-centrosymmetric dimers (*L*)_2_, therefore in the following will be given two values of bond lengths due to comparison of non-coordinated and coordinated *L*.

The planar four-membered metalocycle UONO is characterised by an average angle U—O—N equal to 98.1 °. Bond length U—O(oxido ligand) is equal to 1.754 (3) Å while U—O(NO_3_) and U—O(PO) are 2.523 (3)-2.573 (3) Å and 2.334 (3) Å, respectively. The O—N—O angle of the chelate ring (114.3 (3) °) are shorter as compared to other angles O—N—O (122.3 (4)-123.3 (4) °). N—O(non-coordinated) and N—O(coordinated) distances fall in the range of 1.204 (5) Å and 1.265 (4)-1.270 (5) Å, respectively. In the coordinated *L* ligand the P—O bond length is slightly increased upon coordination (1.492 (3) Å). In the case of the non-coordinated molecule *L* they are 1.479 Å and 1.469 Å. The P—N bond distances between phosphorus atoms and nitrogen atoms of the pyrrolidine substituents are shortened with respect to observed values in *L* (1.613 (4)-1.625 (4) Å) and fall in the range 1.605 (3)-1.613 (4) Å, that can be explained by increasing π-donor bonding in the (N_pyr_)_2_P(O) fragment due to coordination. In 1, the P—N(NH) bond length (1.695 (3) Å) is shortened as compared to (*L*)_2_ (1.697 and 1.707 Å). The C—N distance is increased while C—O distance do not change upon ligand coordination and are equal to 1.359 (4) Å and 1.196 (5) Å, respectively. Angles in the fragment C(O)NHP(O) are slightly changed upon coordination in the range of 2-3°.

### Experimental

The synthesis of *L* was carried out according to the procedure described previously (Znovjyak *et al.*, 2009).

Hydrated nitrate UO_2_(NO_3_)_2_2H_2_O (1 mmol) was solved upon heating in a CH_3_CN (10 ml). The solution was dehydrated by HC(OC_2_H_5_)_3_ (2 mmol), then heated to the boiling point and cooled down. The resulting solution was added to the solution of *L* (2 mmol) in CH_3_OH (10 ml) and was left in a vacuum desiccator over CaCl_2_ at room temperature. After 1 day, the yellow crystals were filtered off and washed with cold isopropanol and dried on the air (yield 80%). IR (KBr, cm^-1^): 3280 ν(NH), 2990 ν(CH), 2890 ν(CH), 1730 ν(CO), 1530, 1440 ν(CN), 1380, 1275, 1215, 1145 ν(PO), 1085, 1030, 940, 890, 820, 680 ν(CCl).

### Refinement

All H atoms were placed at calculated positions and treated as riding on their parent atoms [C—H = 0.93 Å and *U*_iso_(H) = 1.2*U*_eq_(C), N—H = 0.86 Å and *U*_iso_(H) = 1.2*U*_eq_(N)]. In one pyrrolidine group, atoms C3—C6 were treated as disordered between two orientations A and B, respectively, with the occupancies to 0.58 and 0.42.

### Crystal data

**Table:**

[U(NO_3_)_2_O_2_(C_10_H_17_Cl_3_N_3_O_2_P)_2_]	*Z* = 1
*M**_r_* = 1091.22	*F*(000) = 530
Triclinic, *P*1	*D*_x_ = 1.893 Mg m^−^^3^
Hall symbol: -P 1	Mo *K*α radiation, λ = 0.71073 Å
*a* = 9.8292 (7) Å	Cell parameters from 35271 reflections
*b* = 10.3436 (8) Å	θ = 3.0–40.8°
*c* = 10.4475 (6) Å	µ = 4.80 mm^−^^1^
α = 71.905 (6)°	*T* = 293 K
β = 84.391 (5)°	Block, yellow
γ = 71.475 (6)°	0.40 × 0.30 × 0.20 mm
*V* = 957.34 (11) Å^3^	

### Data collection

**Table:**

Oxford Diffraction Xcalibur3 diffractometer	5523 independent reflections
Radiation source: fine-focus sealed tube	5486 reflections with *I* > 2σ(*I*)
graphite	*R*_int_ = 0.090
Detector resolution: 16.1827 pixels mm^-1^	θ_max_ = 30.0°, θ_min_ = 3.0°
ω–scans	*h* = −13→13
Absorption correction: multi-scan (*CrysAlis RED*; Oxford Diffraction, 2006)	*k* = −14→14
*T*_min_ = 0.250, *T*_max_ = 0.447	*l* = −14→14
22849 measured reflections	

### Refinement

**Table:**

Refinement on *F*^2^	Secondary atom site location: difference Fourier map
Least-squares matrix: full	Hydrogen site location: inferred from neighbouring sites
*R*[*F*^2^ > 2σ(*F*^2^)] = 0.034	H-atom parameters constrained
*wR*(*F*^2^) = 0.085	*w* = 1/[σ^2^(*F*_o_^2^) + (0.0645*P*)^2^] where *P* = (*F*_o_^2^ + 2*F*_c_^2^)/3
*S* = 0.98	(Δ/σ)_max_ = 0.001
5523 reflections	Δρ_max_ = 1.93 e Å^−^^3^
260 parameters	Δρ_min_ = −1.68 e Å^−^^3^
56 restraints	Extinction correction: *SHELXL*, Fc^*^=kFc[1+0.001xFc^2^λ^3^/sin(2θ)]^-1/4^
Primary atom site location: structure-invariant direct methods	Extinction coefficient: 0.0164 (15)

### Special details

**Table:**

Geometry. All esds (except the esd in the dihedral angle between two l.s. planes) are estimated using the full covariance matrix. The cell esds are taken into account individually in the estimation of esds in distances, angles and torsion angles; correlations between esds in cell parameters are only used when they are defined by crystal symmetry. An approximate (isotropic) treatment of cell esds is used for estimating esds involving l.s. planes.
Refinement. Refinement of *F*^2^ against ALL reflections. The weighted *R*-factor wR and goodness of fit *S* are based on *F*^2^, conventional *R*-factors *R* are based on *F*, with *F* set to zero for negative *F*^2^. The threshold expression of *F*^2^ > σ(*F*^2^) is used only for calculating *R*-factors(gt) etc. and is not relevant to the choice of reflections for refinement. *R*-factors based on *F*^2^ are statistically about twice as large as those based on *F*, and *R*- factors based on ALL data will be even larger.

### Fractional atomic coordinates and isotropic or equivalent isotropic displacement parameters (Å^2^)

**Table:**

	*x*	*y*	*z*	*U*_iso_*/*U*_eq_	Occ. (<1)
U	0.5000	0.5000	0.5000	0.03209 (7)	
Cl1	1.08718 (12)	0.15464 (16)	0.42224 (11)	0.0674 (3)	
Cl2	1.27581 (13)	−0.08470 (15)	0.60993 (14)	0.0711 (3)	
Cl3	1.23459 (15)	0.1967 (2)	0.6270 (2)	0.0834 (4)	
P1	0.74992 (8)	0.19775 (8)	0.75384 (8)	0.03262 (14)	
O1	0.6348 (3)	0.2904 (3)	0.6531 (3)	0.0474 (6)	
O2	1.0554 (3)	−0.0118 (4)	0.8039 (3)	0.0613 (9)	
O3	0.5489 (3)	0.6026 (4)	0.5826 (3)	0.0526 (6)	
O4	0.6361 (3)	0.5934 (3)	0.2862 (3)	0.0520 (7)	
O5	0.7579 (3)	0.4078 (4)	0.4326 (3)	0.0588 (8)	
O6	0.8601 (4)	0.4932 (4)	0.2460 (4)	0.0688 (10)	
N1	0.8984 (3)	0.1713 (3)	0.6561 (3)	0.0373 (5)	
H1	0.8905	0.2245	0.5738	0.045*	
N3	0.7074 (3)	0.0571 (3)	0.8365 (3)	0.0420 (6)	
N4	0.7559 (3)	0.4981 (4)	0.3181 (3)	0.0432 (6)	
C1	1.0286 (3)	0.0743 (4)	0.6952 (3)	0.0383 (6)	
C2	1.1511 (4)	0.0847 (4)	0.5899 (4)	0.0445 (7)	
N2	0.7919 (3)	0.2608 (4)	0.8625 (4)	0.0450 (6)	
C3A	0.6874 (16)	0.2992 (18)	0.9673 (15)	0.056 (4)	0.58
H3A1	0.7145	0.2300	1.0549	0.067*	0.58
H3A2	0.5915	0.3050	0.9453	0.067*	0.58
C4A	0.6950 (10)	0.4427 (12)	0.9649 (15)	0.072 (3)	0.58
H4A1	0.6628	0.4628	1.0494	0.086*	0.58
H4A2	0.6384	0.5183	0.8917	0.086*	0.58
C5A	0.8571 (12)	0.4237 (17)	0.9427 (18)	0.084 (4)	0.58
H5A1	0.8771	0.5144	0.9155	0.101*	0.58
H5A2	0.9129	0.3609	1.0223	0.101*	0.58
C6A	0.884 (2)	0.355 (2)	0.828 (2)	0.066 (6)	0.58
H6A1	0.8564	0.4270	0.7415	0.079*	0.58
H6A2	0.9840	0.3007	0.8241	0.079*	0.58
C3B	0.692 (3)	0.282 (3)	0.974 (2)	0.075 (7)	0.42
H3B1	0.5935	0.3271	0.9419	0.090*	0.42
H3B2	0.6979	0.1919	1.0426	0.090*	0.42
C4B	0.743 (2)	0.377 (2)	1.0253 (19)	0.096 (5)	0.42
H4B1	0.8132	0.3208	1.0961	0.115*	0.42
H4B2	0.6634	0.4393	1.0611	0.115*	0.42
C5B	0.810 (3)	0.4639 (19)	0.904 (3)	0.096 (6)	0.42
H5B1	0.7379	0.5434	0.8473	0.116*	0.42
H5B2	0.8789	0.4995	0.9322	0.116*	0.42
C6B	0.885 (3)	0.352 (3)	0.831 (3)	0.072 (8)	0.42
H6B1	0.8895	0.3961	0.7349	0.086*	0.42
H6B2	0.9811	0.2988	0.8655	0.086*	0.42
C7	0.6093 (4)	0.0065 (5)	0.7792 (4)	0.0497 (8)	
H7A	0.6362	0.0058	0.6875	0.060*	
H7B	0.5109	0.0666	0.7797	0.060*	
C8	0.6270 (8)	−0.1427 (7)	0.8705 (8)	0.0852 (18)	
H8A	0.5426	−0.1457	0.9265	0.102*	
H8B	0.6408	−0.2085	0.8180	0.102*	
C9	0.7537 (12)	−0.1812 (7)	0.9542 (8)	0.109 (3)	
H9A	0.8378	−0.2340	0.9145	0.131*	
H9B	0.7402	−0.2411	1.0435	0.131*	
C10	0.7735 (5)	−0.0494 (5)	0.9627 (4)	0.0582 (10)	
H10A	0.8744	−0.0588	0.9669	0.070*	
H10B	0.7250	−0.0240	1.0409	0.070*	

### Atomic displacement parameters (Å^2^)

**Table:**

	*U*^11^	*U*^22^	*U*^33^	*U*^12^	*U*^13^	*U*^23^
U	0.02715 (9)	0.02741 (9)	0.03229 (10)	−0.00624 (5)	−0.00343 (5)	0.00313 (5)
Cl1	0.0505 (5)	0.0857 (9)	0.0435 (5)	−0.0017 (5)	0.0060 (4)	−0.0091 (5)
Cl2	0.0527 (6)	0.0636 (7)	0.0744 (7)	0.0138 (5)	0.0040 (5)	−0.0225 (6)
Cl3	0.0579 (6)	0.0868 (10)	0.1247 (13)	−0.0366 (7)	0.0060 (7)	−0.0446 (10)
P1	0.0271 (3)	0.0258 (3)	0.0332 (3)	−0.0036 (2)	−0.0009 (2)	0.0033 (3)
O1	0.0287 (10)	0.0412 (13)	0.0442 (12)	0.0003 (9)	−0.0008 (9)	0.0152 (10)
O2	0.0430 (14)	0.067 (2)	0.0440 (14)	0.0099 (13)	−0.0099 (11)	0.0004 (14)
O3	0.0576 (16)	0.0518 (17)	0.0555 (16)	−0.0269 (13)	−0.0045 (12)	−0.0136 (13)
O4	0.0400 (12)	0.0461 (15)	0.0446 (13)	−0.0036 (11)	0.0033 (10)	0.0110 (11)
O5	0.0386 (13)	0.0545 (18)	0.0472 (14)	0.0013 (11)	0.0060 (11)	0.0173 (12)
O6	0.0469 (15)	0.072 (2)	0.0606 (18)	−0.0101 (15)	0.0194 (14)	0.0037 (16)
N1	0.0283 (11)	0.0379 (14)	0.0335 (12)	−0.0001 (9)	−0.0020 (9)	−0.0032 (10)
N3	0.0400 (13)	0.0332 (14)	0.0412 (14)	−0.0116 (11)	−0.0122 (11)	0.0093 (11)
N4	0.0355 (13)	0.0411 (15)	0.0393 (14)	−0.0073 (11)	0.0042 (10)	0.0016 (11)
C1	0.0307 (13)	0.0410 (17)	0.0385 (14)	−0.0019 (11)	−0.0061 (11)	−0.0124 (13)
C2	0.0318 (14)	0.0473 (19)	0.0478 (18)	−0.0044 (13)	−0.0006 (12)	−0.0124 (15)
N2	0.0428 (14)	0.0428 (16)	0.0518 (16)	−0.0165 (12)	0.0106 (12)	−0.0170 (14)
C3A	0.052 (6)	0.067 (7)	0.056 (6)	−0.023 (5)	0.034 (6)	−0.035 (6)
C4A	0.049 (4)	0.071 (6)	0.106 (8)	−0.009 (4)	0.013 (5)	−0.054 (6)
C5A	0.054 (5)	0.094 (9)	0.141 (11)	−0.028 (6)	0.021 (6)	−0.087 (9)
C6A	0.055 (10)	0.064 (10)	0.107 (12)	−0.043 (8)	0.032 (9)	−0.049 (9)
C3B	0.073 (13)	0.066 (11)	0.082 (14)	−0.011 (8)	−0.019 (9)	−0.024 (9)
C4B	0.112 (13)	0.102 (13)	0.096 (11)	−0.030 (10)	0.004 (9)	−0.064 (10)
C5B	0.109 (16)	0.059 (9)	0.134 (14)	−0.019 (9)	0.002 (11)	−0.055 (9)
C6B	0.055 (14)	0.066 (15)	0.100 (15)	−0.006 (10)	−0.007 (9)	−0.042 (12)
C7	0.0418 (17)	0.052 (2)	0.054 (2)	−0.0214 (16)	−0.0004 (14)	−0.0055 (17)
C8	0.089 (4)	0.058 (3)	0.110 (5)	−0.046 (3)	−0.009 (3)	0.000 (3)
C9	0.171 (8)	0.042 (3)	0.102 (5)	−0.043 (4)	−0.060 (5)	0.024 (3)
C10	0.059 (2)	0.048 (2)	0.0467 (19)	−0.0157 (18)	−0.0118 (16)	0.0179 (16)

### Geometric parameters (Å, °)

**Table:**

U—O3	1.754 (3)	C4A—C5A	1.546 (12)
U—O3^i^	1.754 (3)	C4A—H4A1	0.9700
U—O1	2.334 (2)	C4A—H4A2	0.9700
U—O1^i^	2.334 (2)	C5A—C6A	1.538 (13)
U—O5	2.523 (3)	C5A—H5A1	0.9700
U—O5^i^	2.523 (3)	C5A—H5A2	0.9700
U—O4^i^	2.571 (3)	C6A—H6A1	0.9700
U—O4	2.571 (3)	C6A—H6A2	0.9700
Cl1—C2	1.768 (4)	C3B—C4B	1.482 (16)
Cl2—C2	1.753 (4)	C3B—H3B1	0.9700
Cl3—C2	1.764 (4)	C3B—H3B2	0.9700
P1—O1	1.491 (2)	C4B—C5B	1.542 (18)
P1—N3	1.604 (3)	C4B—H4B1	0.9700
P1—N2	1.615 (3)	C4B—H4B2	0.9700
P1—N1	1.696 (3)	C5B—C6B	1.531 (15)
O2—C1	1.197 (5)	C5B—H5B1	0.9700
O4—N4	1.267 (4)	C5B—H5B2	0.9700
O5—N4	1.265 (4)	C6B—H6B1	0.9700
O6—N4	1.208 (4)	C6B—H6B2	0.9700
N1—C1	1.357 (4)	C7—C8	1.507 (8)
N1—H1	0.8600	C7—H7A	0.9700
N3—C10	1.478 (5)	C7—H7B	0.9700
N3—C7	1.481 (5)	C8—C9	1.468 (10)
C1—C2	1.556 (5)	C8—H8A	0.9700
N2—C6B	1.460 (14)	C8—H8B	0.9700
N2—C6A	1.475 (8)	C9—C10	1.467 (8)
N2—C3B	1.473 (14)	C9—H9A	0.9700
N2—C3A	1.484 (7)	C9—H9B	0.9700
C3A—C4A	1.502 (13)	C10—H10A	0.9700
C3A—H3A1	0.9700	C10—H10B	0.9700
C3A—H3A2	0.9700		
			
O3—U—O3^i^	180.00 (13)	C4A—C3A—H3A2	111.1
O3—U—O1	90.47 (14)	H3A1—C3A—H3A2	109.0
O3^i^—U—O1	89.53 (14)	C3A—C4A—C5A	101.7 (8)
O3—U—O1^i^	89.53 (14)	C3A—C4A—H4A1	111.4
O3^i^—U—O1^i^	90.47 (14)	C5A—C4A—H4A1	111.4
O1—U—O1^i^	180.0	C3A—C4A—H4A2	111.4
O3—U—O5	90.00 (14)	C5A—C4A—H4A2	111.4
O3^i^—U—O5	90.00 (14)	H4A1—C4A—H4A2	109.3
O1—U—O5	65.33 (9)	C6A—C5A—C4A	99.5 (10)
O1^i^—U—O5	114.67 (9)	C6A—C5A—H5A1	111.9
O3—U—O5^i^	90.00 (14)	C4A—C5A—H5A1	111.9
O3^i^—U—O5^i^	90.00 (14)	C6A—C5A—H5A2	111.9
O1—U—O5^i^	114.67 (9)	C4A—C5A—H5A2	111.9
O1^i^—U—O5^i^	65.33 (9)	H5A1—C5A—H5A2	109.6
O5—U—O5^i^	180.0	N2—C6A—C5A	103.7 (8)
O3—U—O4^i^	88.35 (13)	N2—C6A—H6A1	111.0
O3^i^—U—O4^i^	91.65 (13)	C5A—C6A—H6A1	111.0
O1—U—O4^i^	65.33 (8)	N2—C6A—H6A2	111.0
O1^i^—U—O4^i^	114.67 (8)	C5A—C6A—H6A2	111.0
O5—U—O4^i^	130.60 (9)	H6A1—C6A—H6A2	109.0
O5^i^—U—O4^i^	49.40 (9)	C4B—C3B—N2	102.4 (11)
O3—U—O4	91.65 (13)	C4B—C3B—H3B1	111.3
O3^i^—U—O4	88.35 (13)	N2—C3B—H3B1	111.3
O1—U—O4	114.67 (8)	C4B—C3B—H3B2	111.3
O1^i^—U—O4	65.33 (8)	N2—C3B—H3B2	111.3
O5—U—O4	49.40 (9)	H3B1—C3B—H3B2	109.2
O5^i^—U—O4	130.60 (9)	C3B—C4B—C5B	105.9 (13)
O4^i^—U—O4	180.0	C3B—C4B—H4B1	110.6
O1—P1—N3	108.41 (16)	C5B—C4B—H4B1	110.6
O1—P1—N2	119.57 (19)	C3B—C4B—H4B2	110.6
N3—P1—N2	107.23 (17)	C5B—C4B—H4B2	110.6
O1—P1—N1	102.49 (14)	H4B1—C4B—H4B2	108.7
N3—P1—N1	115.72 (16)	C6B—C5B—C4B	101.6 (13)
N2—P1—N1	103.76 (15)	C6B—C5B—H5B1	111.5
P1—O1—U	157.1 (2)	C4B—C5B—H5B1	111.5
N4—O4—U	96.89 (19)	C6B—C5B—H5B2	111.5
N4—O5—U	99.3 (2)	C4B—C5B—H5B2	111.5
C1—N1—P1	126.3 (2)	H5B1—C5B—H5B2	109.3
C1—N1—H1	116.9	N2—C6B—C5B	102.6 (11)
P1—N1—H1	116.9	N2—C6B—H6B1	111.2
C10—N3—C7	110.9 (3)	C5B—C6B—H6B1	111.2
C10—N3—P1	127.0 (3)	N2—C6B—H6B2	111.2
C7—N3—P1	121.4 (2)	C5B—C6B—H6B2	111.2
O6—N4—O5	122.5 (3)	H6B1—C6B—H6B2	109.2
O6—N4—O4	123.1 (3)	N3—C7—C8	104.1 (4)
O5—N4—O4	114.4 (3)	N3—C7—H7A	110.9
O2—C1—N1	125.1 (3)	C8—C7—H7A	110.9
O2—C1—C2	119.3 (3)	N3—C7—H7B	110.9
N1—C1—C2	115.5 (3)	C8—C7—H7B	110.9
C1—C2—Cl2	109.9 (3)	H7A—C7—H7B	108.9
C1—C2—Cl3	105.5 (2)	C9—C8—C7	106.4 (4)
Cl2—C2—Cl3	109.3 (2)	C9—C8—H8A	110.4
C1—C2—Cl1	112.6 (2)	C7—C8—H8A	110.4
Cl2—C2—Cl1	108.7 (2)	C9—C8—H8B	110.4
Cl3—C2—Cl1	110.8 (2)	C7—C8—H8B	110.4
C6B—N2—C6A	1(2)	H8A—C8—H8B	108.6
C6B—N2—C3B	113.4 (9)	C8—C9—C10	108.4 (5)
C6A—N2—C3B	113.9 (9)	C8—C9—H9A	110.0
C6B—N2—C3A	109.0 (9)	C10—C9—H9A	110.0
C6A—N2—C3A	109.3 (6)	C8—C9—H9B	110.0
C3B—N2—C3A	6.4 (17)	C10—C9—H9B	110.0
C6B—N2—P1	123.2 (10)	H9A—C9—H9B	108.4
C6A—N2—P1	122.1 (7)	C9—C10—N3	103.0 (4)
C3B—N2—P1	118.4 (8)	C9—C10—H10A	111.2
C3A—N2—P1	120.3 (6)	N3—C10—H10A	111.2
N2—C3A—C4A	103.5 (7)	C9—C10—H10B	111.2
N2—C3A—H3A1	111.1	N3—C10—H10B	111.2
C4A—C3A—H3A1	111.1	H10A—C10—H10B	109.1
N2—C3A—H3A2	111.1		

Symmetry codes: (i) −*x*+1, −*y*+1, −*z*+1.

### Hydrogen-bond geometry (Å, °)

**Table:**

*D*—H···*A*	*D*—H	H···*A*	*D*···*A*	*D*—H···*A*
N1—H1···O5	0.86	2.13	2.877 (4)	145
